# Interference of Intravenous Acetaminophen with Continuous Glucose Monitoring System

**DOI:** 10.31662/jmaj.2025-0186

**Published:** 2025-09-12

**Authors:** Misayo Matsuyama, Satoru Meiri, Hirotake Sawada, Ryuta Masuya, Kazuhiko Nakame, Hiroshi Moritake

**Affiliations:** 1 Division of Pediatrics, Faculty of Medicine, University of Miyazaki, Miyazaki, Japan; 2 Department of Fundamental Nursing, Faculty of Medicine, University of Miyazaki, Miyazaki, Japan; 3 Department of Surgery, Division of the Gastrointestinal, Endocrine and Pediatric Surgery, Faculty of Medicine, University of Miyazaki, Miyazaki, Japan

**Keywords:** automated insulin delivery, sensor-augmented pump, intravenous acetaminophen, continuous glucose monitoring

## Abstract

Sensor-augmented pumps (SAPs) and automated insulin delivery (AID) systems are innovative technologies for diabetes management. Accurate continuous glucose monitoring (CGM) is crucial for their safe and effective use; however, certain commonly used drugs can interfere with CGM accuracy. Although acetaminophen is known to cause falsely elevated CGM glucose values, previous CGM studies have primarily focused on its oral administration, with limited data on intravenous use. We report a case of a CGM reaction after the intravenous administration of acetaminophen in a boy with type 1 diabetes using SAP. The patient received repeated doses of intravenous acetaminophen (15 mg/kg for 15 min) for pain relief. After administration, we recorded a rapid increase in his CGM readings without a corresponding increase in blood glucose levels. The CGM glucose peaked at 29.2 ± 1.9 min (mean ± standard deviation) after administration and an estimated discrepancy of 55 to 114 mg/dL compared with capillary blood glucose measurements. Discrepancies between measured blood glucose and CGM readings were significantly greater at lower glucose levels. These falsely elevated CGM readings could potentially trigger an inappropriate autocorrection bolus in AID systems and increase the risk of hypoglycemia. Medical professionals should be fully aware of acetaminophen-induced CGM interference, particularly the potential risks in patients using AID systems.

## Introduction

Technologies for diabetes care have advanced significantly. The development of sensor-augmented pumps (SAPs) linked to continuous glucose monitoring (CGM) systems, particularly automated insulin delivery (AID) systems that automatically infuse insulin using an advanced algorithm, is expected to facilitate significant blood glucose control ^(1), (2)^. Accurate CGM is critical for these systems; however, certain drugs can affect this accuracy. Acetaminophen is known to cause falsely elevated CGM values, with previous CGM studies focusing on oral acetaminophen ^(3), (4)^. We report a case of a significant CGM misreading in a child with type 1 diabetes after intravenous acetaminophen administration to alert medical professionals about this potential interference.

## Case Report

**Figure 1.**
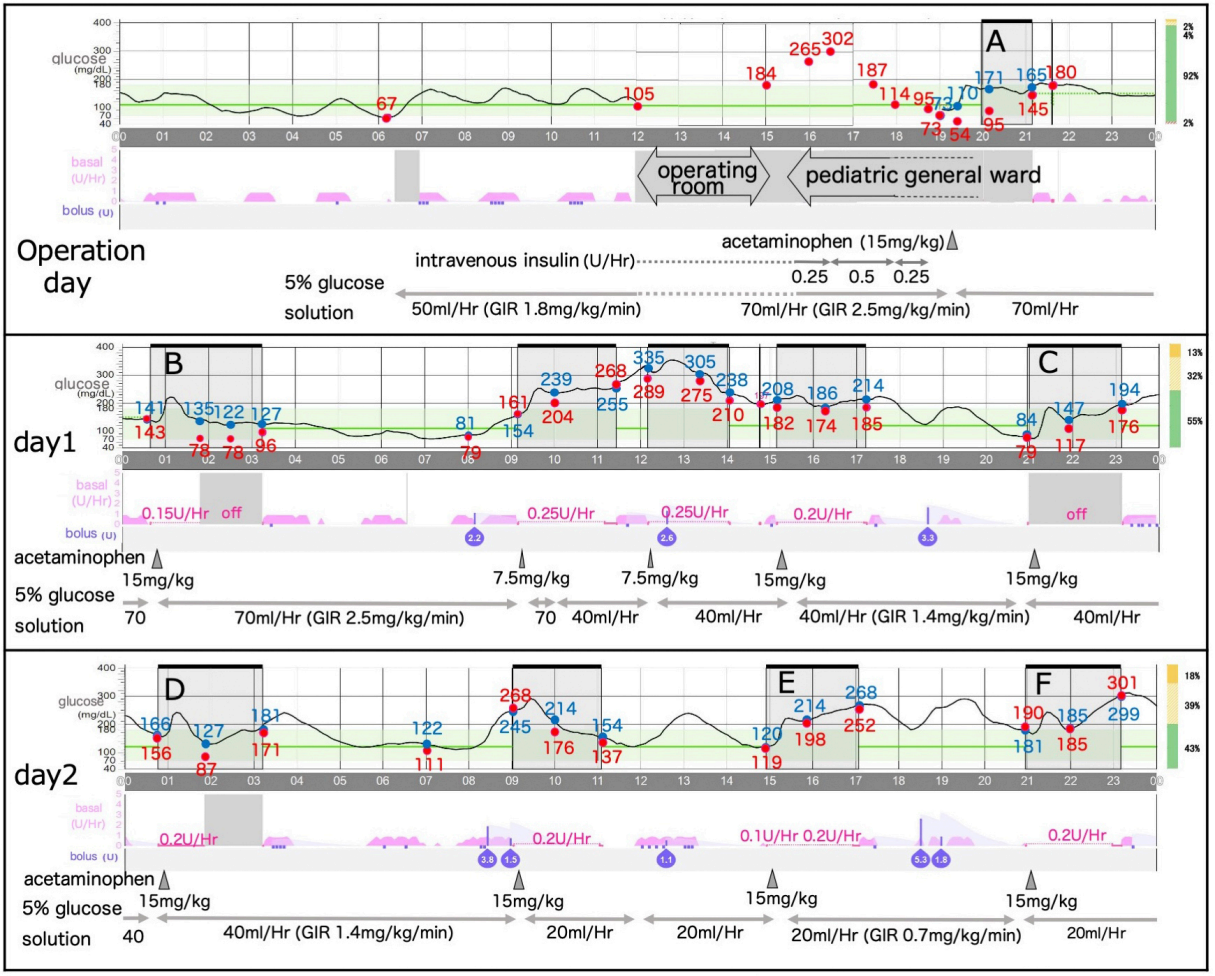
Overview of blood glucose and CGM glucose and insulin injection. Red circles indicate blood glucose levels, and blue circles indicate CGM glucose readings. CGM: continuous glucose monitoring; GIR: glucose infusion rate.

The patient was a 7-year-old boy with type 1 diabetes, using an AID system (MiniMed 780G insulin pump and a Guardian 4 sensor [Medtronic Inc., Dublin, Ireland]). He was admitted for an elective laparoscopic appendectomy. At the patient’s and family’s request, the AID system was used during hospitalization, with the pump and CGM sensor removed only during surgery. Postoperatively, the insulin pump was initiated in the hybrid closed-loop (HCL) mode with a temporary target of 150 mg/dL. One day after surgery, the HCL mode was switched to the advanced hybrid closed-loop mode with a target of 120 mg/dL. The active insulin time was set to 2 hours, consistent with standard use. Bedside blood glucose checks were performed using the ACCU-CHEK Guide Link (Roche, Basel, Switzerland) and Nipro Stat Strip XP3 (Nova Biomedical Corp., Boston, MA, USA). Both devices were used when acetaminophen was initially administered and exhibited no significant difference; therefore, only the ACCU-CHEK Guide Link was used during subsequent acetaminophen administration. Post surgery, acetaminophen (15 mg/kg/dose, intravenous [IV]) was administered every 6 hours for pain relief. During acetaminophen administration, the pump was switched to manual mode to prevent automatic insulin infusion. After the first dose, the CGM reading increased rapidly, forming an upward convex curve. One hour later, the patient’s measured blood glucose was 95 mg/dL, whereas the CGM reported 171 mg/dL and maintained a discrepancy for more than 2 hours (Figure 1). After the discrepancy decreased, the pump system was switched to HCL mode. A similar blood glucose pattern was observed after subsequent administrations, and the insulin pump was managed as previously described (Figure 1).

**Figure 2.**
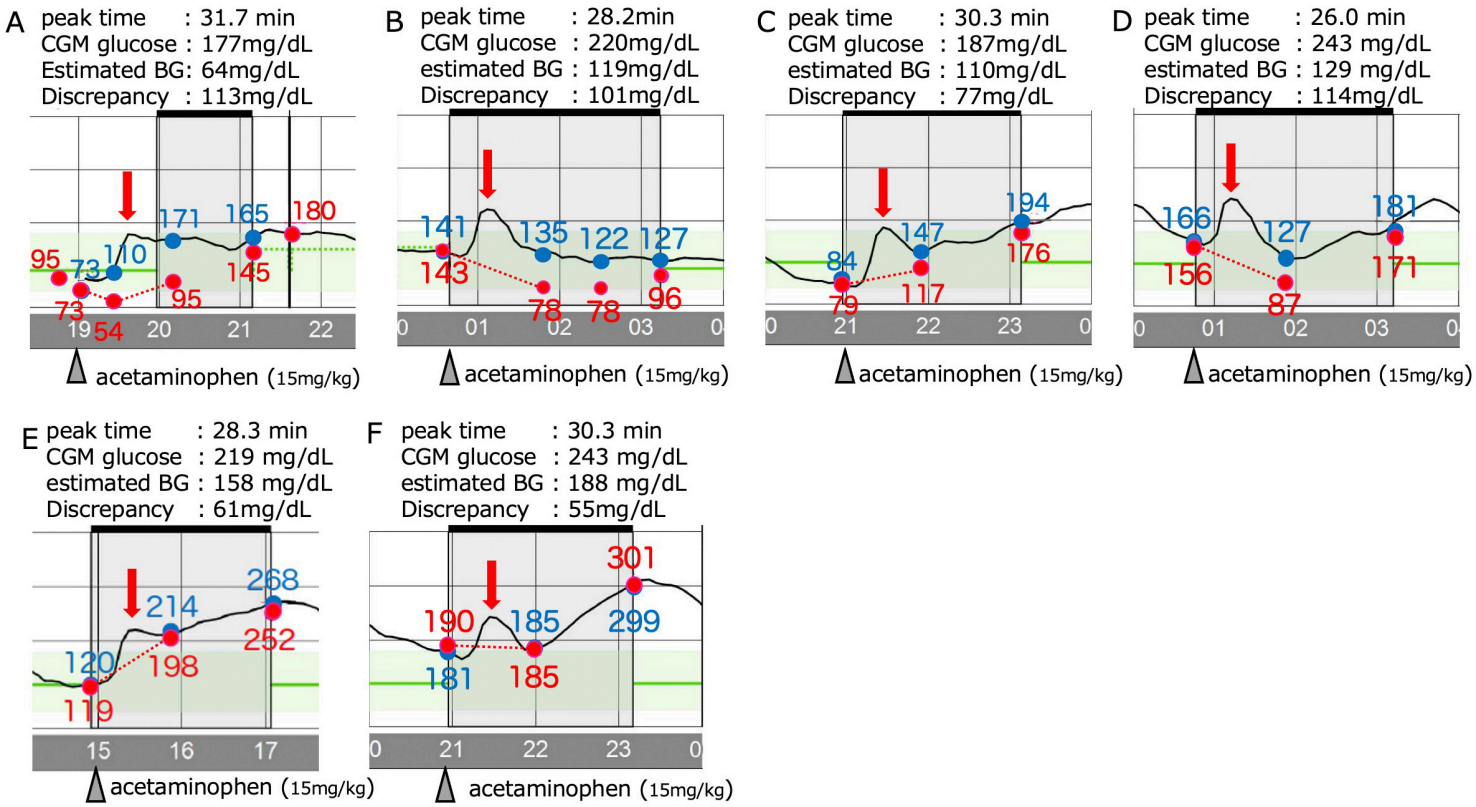
Glucose discrepancy between blood measurements and CGM readings after acetaminophen administration. Red arrows indicate peak CGM glucose after acetaminophen administration. Blood glucose levels were measured assuming that they varied linearly (red dotted line). BG: blood glucose; CGM: continuous glucose monitoring.

**Figure 3.**
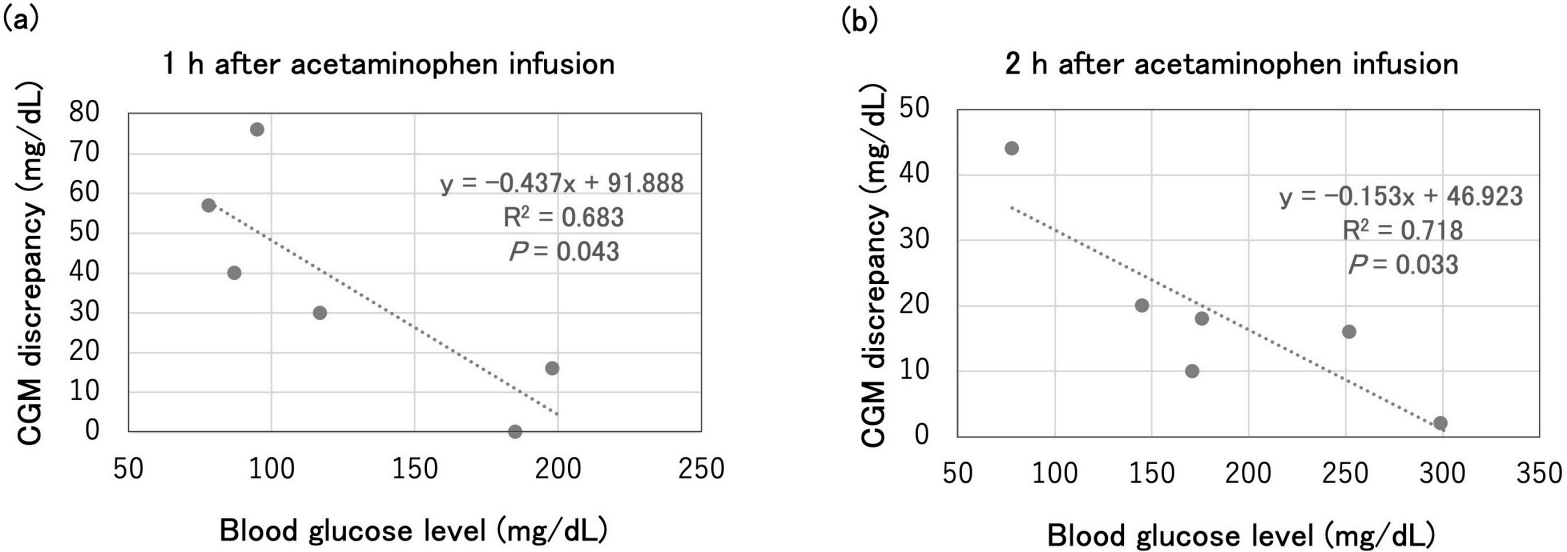
Analysis of blood glucose levels and CGM discrepancies after acetaminophen administration. Statistical significance was evaluated to calculate the *p*-value, and goodness-of-fit was tested using the R^2^ value. Differences were considered statistically significant at p < 0.05. CGM: continuous glucose monitoring.

We extracted CGM data from the insulin pump. We selected six occasions (Figure 1A-F) on which oral intake did not occur and when no significant insulin bolus injections were performed for at least 2 hours before acetaminophen. We quantified the CGM readings and determined peak CGM glucose levels and the corresponding times after giving intravenous acetaminophen using WebPlotDigitizer 4.8 (https://automeris.io/WebPlotDigitizer). Because the insulin dosage remained constant from the start of acetaminophen administration, we assumed that blood glucose changed linearly. We formulated linear equations based on blood glucose levels and time points at the start of acetaminophen administration and 1 hour after administration and calculated the estimated glucose levels at the CGM peak times (Figure 2). The CGM glucose readings peaked at 29.2 ± 1.9 min (mean ± standard deviation [SD]), after acetaminophen administration (15 mg/kg over 15 min IV), with the estimated CGM discrepancy ranging from 55 to 114 mg/dL (Figure 2). Subsequently, we conducted a linear regression analysis to assess the relationship between blood glucose levels and CGM discrepancies after acetaminophen administration, using R software (version 4.4.1) (https://www.r-project.org/). The analysis was conducted at 12 time points from the previously selected six occasions, using paired data comprising measured glucose values and corresponding CGM readings at 1 hour and 2 hours after the administration of 15 mg/kg of acetaminophen. We found a significant negative correlation between blood glucose levels and CGM discrepancies 1 and 2 hours after acetaminophen administration (i.e., as blood glucose levels decreased, the discrepancy increased; Figure 3). A similar trend was observed with the six estimated maximum discrepancies shown in Figure 2; however, the difference was not statistically significant (data not shown).

## Discussion

Acetaminophen has been reported to cause falsely elevated CGM readings ^(3), (4)^. Medtronic and Dexcom CGM sensors measure glucose concentrations by monitoring the electrical signals generated from the glucose oxidation reaction. When interstitial glucose passes through the glucose oxidase membrane, hydrogen peroxide (H_2_O_2_) is produced. Under an applied voltage, H_2_O_2_ decomposes and releases electrons, which are converted into blood glucose values. However, because this reaction requires high voltage, easily oxidized substances such as acetaminophen and ascorbic acid can interfere with glucose detection ^(5)^. After oral administration of 1 g of acetaminophen, CGM readings increased from baseline by 21 mg/dL in the Guardian REAL-TIME system and by 30 mg/dL in the Dexcom G4 Platinum system, under plasma glucose conditions of approximately 90 mg/dL ^(3)^. With the more recent Dexcom G6 system, the mean discrepancy between CGM and plasma glucose increased by 3.1 mg/dL (SD ± 4.8 mg/dL) from baseline after a 1 g oral dose of acetaminophen ^(6)^; however, reports of the Guardian 4 sensor are not available. Furthermore, to the best of our knowledge, no studies have examined the effects of intravenous acetaminophen administration on CGM accuracy.

In this case, intravenous acetaminophen (15 mg/kg for 15 minutes) caused a sharp increase in CGM readings and a peak at approximately 30 minutes. The estimated discrepancy between the CGM system and measured glucose at peak time ranged from 55 to 114 mg/dL. These false elevated CGM readings were greater than those we had anticipated on the basis of previous reports ^(3), (6)^. This may be due to the intravenous route, which produces approximately twice the blood concentration of acetaminophen than does oral administration ^(7)^, which potentially causes significant CGM inaccuracies.

In the analysis of CGM readings and measured glucose levels, a negative correlation was observed at 1 hour after acetaminophen administration, with greater discrepancies at lower glucose levels. Although the discrepancy decreased at 2 hours after administration, this negative correlation persisted. These findings indicate that the effect of acetaminophen on CGM readings is greater and more prolonged at lower blood glucose levels.

To our knowledge, this is the first report of intravenous acetaminophen affecting CGM readings. These falsely elevated CGM glucose values may trigger unnecessary autocorrection boluses by the AID system and increase the risk of hypoglycemia. Furthermore, the association between lower blood glucose levels and greater CGM discrepancies suggests an elevated hypoglycemia risk in users of AID receiving intravenous acetaminophen.

Although acetaminophen interference with CGM is well-known and should be avoided, intravenous acetaminophen is often the only option for postoperative pain relief for children. Switching from daily AID to manual mode may impair glucose control and require frequent glucose monitoring, thus increasing the patient burden. Therefore, we only used the manual mode when the effect of acetaminophen was significant, and switched to the automatic mode once this effect diminished. This approach successfully prevented hypoglycemia; however, given responses to acetaminophen may vary depending on the individual and clinical factors, management should be tailored to each case. This case emphasizes that medical professionals should be aware of the potential interference of acetaminophen with CGM accuracy and particularly its risk for users of AID systems.

## Article Information

### Author Contributions

Misayo Matsuyama cared for the patients and was involved in writing, designing, and editing the manuscript. Satoru Meiri, Ryuta Masuya, and Kazuhiko Nakame cared for the patients and edited the manuscript. Hirotake Sawada and Hiroshi Moritake supported the statistical analyses and edited the manuscript. All the authors have read and approved the published version of the manuscript.

### Conflicts of Interest

None

### IRB Approval Code and Name of the Institution

Not applicable. Informed consent was obtained from the parents of a patient.
